# Increased Maternal Prenatal Adiposity, Inflammation, and Lower Omega-3 Fatty Acid Levels Influence Child Negative Affect

**DOI:** 10.3389/fnins.2019.01035

**Published:** 2019-10-01

**Authors:** Hanna C. Gustafsson, Kathleen F. Holton, Ashley N. Anderson, Elizabeth K. Nousen, Ceri A. Sullivan, Jennifer M. Loftis, Joel T. Nigg, Elinor L. Sullivan

**Affiliations:** ^1^Department of Psychiatry, Oregon Health and Science University, Portland, OR, United States; ^2^Department of Health Studies, Center for Behavioral Neuroscience, American University, Washington, DC, United States; ^3^VA Portland Health Care System, Portland, OR, United States; ^4^Department of Behavioral Neuroscience, Oregon Health and Science University, Portland, OR, United States; ^5^Division of Neuroscience, Oregon National Primate Research Center, Beaverton, OR, United States; ^6^Department of Human Physiology, University of Oregon, Eugene, OR, United States

**Keywords:** pre-pregnancy body mass index, omega-3 fatty acids, DHA, EPA, infant temperament, negative affect, inflammation

## Abstract

**Objective:**

Increased maternal adiposity during pregnancy is associated with offspring risk for psychiatric disorders. Inflammation secondary to adiposity is believed to be an important mechanism through which this effect occurs. Although increased adiposity introduces risk, not all children of overweight mothers develop these problems. Gestational factors that modify this risk are not well-understood. If maternal increased adiposity exerts its effects on offspring outcomes by increasing inflammation in the gestational environment, then anti-inflammatory inputs such as omega-3 fatty acids may be one protective factor. The goal of this study was to investigate whether maternal pre-pregnancy body mass index (BMI) and omega-3 fatty acid levels independently and/or interactively predicted offspring infant negative affect, an early life marker of risk for psychopathology.

**Methods:**

Data came from a prospective study of women recruited during pregnancy and their 6 month old infants (*N* = 62; 40% female). Maternal pre-pregnancy BMI was pulled from medical charts and third trimester omega-3 fatty acid concentrations were assessed in plasma. Child negative affect was assessed using observer- and maternal-ratings at 6 months of age. Maternal inflammation was indexed by third trimester plasma levels of interleukin-6, tumor necrosis factor-alpha, and monocyte chemoattractant protein-1.

**Results:**

Maternal pre-pregnancy BMI was associated with increased infant negative affect whereas eicosapentaenoic acid was associated with less infant negative affect. Maternal omega-3 fatty acid levels moderated the effect of BMI on infant negative affect, such that omega-3 fatty acids buffered children against the negative consequences of increased adiposity. Supporting the role of maternal inflammation in these associations, maternal BMI and omega-3 fatty acid levels interacted to predict maternal third trimester inflammation. Further, maternal inflammation was associated with increased infant negative affect.

**Conclusion:**

Results suggest that omega-3 supplementation during pregnancy may protect against offspring behavioral risk associated with increased maternal adiposity.

## Introduction

Increased maternal adiposity in the prenatal period introduces risks to maternal and obstetric health (Schmatz et al., 2010; Marshall and Spong, 2012) but also to the developing child (Catalano and Ehrenberg, 2006; Poston, 2012; Menting et al., 2018). The potential public health importance of these risks is exceptional because a significant proportion of pregnant women in the United States are overweight or obese (King, 2006; Huda et al., 2010). Inflammation secondary to adiposity is believed to be an important mechanism through which this effect occurs. Increased adipocyte mass releases a host of inflammatory cytokines, which increase the *in utero* inflammatory profile and alter fetal brain development in ways that contribute to the offspring’s long-term mood and behavior (Bilbo and Tsang, 2010; Schmatz et al., 2010). Indeed, higher maternal pre-pregnancy body mass index (BMI) is associated with risk for neurodevelopmental and psychiatric disorders in offspring, including attention-deficit/hyperactivity disorder (ADHD), anxiety, and depression (Rivera et al., 2015; Edlow, 2016). These disorders are thought to be rooted in early development. Their risk appears to be detectable in part by increased behavioral and emotional dysregulation in infancy, for example by increased infant negative affect (NA) (the propensity to feel and express negative emotions, i.e., increased crying, fearfulness, and fear reactivity) (Nigg, 2006; Rothbart, 2007). Few studies, however, have examined early markers of behavioral risk in relation to maternal adiposity. Doing so holds promise to (a) inform our understanding of the developmental mechanisms through which increased maternal BMI shapes offspring risk for future neurodevelopmental and psychological disorders, and (b) provide clarity as to when in development this risk appears and thus can be intervened upon.

Over the past decade, members of our research team have developed a non-human primate model to study the effects of maternal obesity on offspring behaviors that serve as analogs for psychiatric disorders. In this model, adult female macaques are fed either a western-style diet (WSD), comparable in fat content to the average contemporary American diet, or a control diet for at least 2 years prior to pregnancy, throughout pregnancy, and during the lactation period. As expected, the WSD promoted weight gain and increased adiposity in most animals. We discovered a series of long-lasting alterations in the behavior of offspring from mothers consuming the WSD (Sullivan et al., 2010; Thompson et al., 2017). These behavioral alterations, which were primarily in the domain of negative valence systems, were due to both the WSD and to increased maternal adiposity associated with that diet (Thompson et al., 2018). Both male and female offspring showed increased anxiety-like behaviors (Sullivan et al., 2010; Thompson et al., 2017). Supporting the hypothesis that increased inflammation may be part of the mechanism through which these effects occur, we recently reported that maternal pre-pregnancy adiposity was associated with increased gestational inflammation, which in turn was associated with offspring behaviors indicative of anxiety (Thompson et al., 2018).

These results are noteworthy for two reasons. First, they offer proof-of-concept that the effects of maternal obesity on offspring behavioral and emotional dysregulation can be detected early in development. Second, they demonstrate that maternal diet and weight can exert independent effects on offspring behavioral development. The latter is commonly assumed, but not often tested, particularly with regard to offspring behavioral outcomes. Whether these novel components of this research translate to human populations remains relatively untested. The role that specific nutrients such as individual fatty acids (FAs) play in programing offspring behavior is also unclear.

Though maternal obesity appears to place children at risk for psychological and behavioral problems, not all children of overweight or obese mothers develop such difficulties. Given how common maternal obesity currently is, the determination of gestational factors that protect offspring from alterations in neurodevelopment programed by maternal obesity is crucial. If maternal obesity exerts its effects on offspring outcomes by increasing inflammation in the gestational environment (Bilbo and Tsang, 2010; Segovia et al., 2014) then anti-inflammatory inputs, such as circulating omega-3 (n-3) FAs, may ameliorate these effects. For example, the fetus of a woman who is overweight (a pro-inflammatory state) but who has high levels of n-3 FAs (an anti-inflammatory input) may be exposed to less gestational inflammation than one whose mother is overweight and has fewer circulating n-3 FAs. These differences in exposure to inflammation may underlie differences in offspring neurobehavioral outcomes.

Omega-3 FA supplementation effectively reduces inflammation and inflammation-associated sequelae in the context of autoimmune diseases (Simopoulos, 2002; Calder, 2007) as well as several psychiatric conditions (Freeman et al., 2006; McNamara and Carlson, 2006); whether these anti-inflammatory effects extend to inflammation secondary to adiposity has not yet been tested, but is theoretically plausible. Preliminary research with rodents prenatally exposed to maternal immune activation supports the idea that n-3 supplementation may buffer offspring from the behavioral consequences of such exposure. Offspring exposed to gestational inflammation (induced via maternal immune activation) fed an n-3 supplemented diet exhibited less anxiety than those who did not receive such supplementation (Li et al., 2015).

The goal of the current study was to examine the independent and interactive effects of maternal pre-pregnancy BMI and circulating n-3 FAs on infant NA, an early life risk factor for psychopathology. To align our human study with those we previously conducted in non-human primates, we focus on indicators of infant fear and sadness (dimensions of NA thought to underlie the development of anxiety) assessed at 6 months of age (to align with the non-human primate studies).

Our research questions were: (1) *Are maternal pre-pregnancy BMI and maternal third trimester circulating n-3 FAs associated with infant NA at 6 months of age?* (2) *Are these effects independent of one another?* (3) *Do maternal pre-pregnancy BMI and maternal circulating n-3 FAs interact to predict infant NA?* We hypothesized that maternal pre-pregnancy BMI and gestational n-3 FAs would be independently associated with infant NA (such that increased BMI would be associated with more NA and higher levels of gestational n-3 FAs with less NA). Additionally, we hypothesized that BMI and n-3 FAs would interact to predict infant NA, with higher n-3 FAs serving as a protective factor.

As described, maternal BMI and FAs are purported to be linked with infant NA via alterations in maternal systemic inflammation during pregnancy. An explicit test of this mechanism was outside of the scope of the current study (largely due to this study’s relatively small sample size and its incompatibility with the complexity of the statistical model that would be needed to test this mechanism explicitly). However, we did conduct initial analyses, to provide additional context for our findings and preliminary proof of concept.

## Materials and Methods

### Procedure

Data used in this study came from an ongoing longitudinal pilot study described previously (Sullivan et al., 2015). Pregnant women were recruited in the second trimester and followed into the postpartum period. Data used in the current analyses came from laboratory visits that occurred at 37 weeks gestation and at 6 months postpartum. During the 37-week visit, women provided a blood sample and completed questionnaire measures of their mood and demographic information. When their child was 6 months old, mother-infant dyads completed a laboratory visit that included several behavioral assessments. Women also completed questionnaires about their infant’s behavior. This laboratory visit was approximately 90 min and was videotaped for later coding. The testing room was equipped with a high chair, a chair for the mother, and two cameras (one focused on the infant, one on the mother). Participants’ medical records were reviewed for information about maternal health, including pre-pregnancy BMI. Oregon Health and Science University’s (OHSU) Institutional Review Board approved all procedures, and written informed consent was obtained from all participants.

### Participants

#### Recruitment

Pregnant women were recruited from OHSU during their second trimester. In enrolling participants, an effort was made to over-select families likely to have offspring with a full range of emotion and behavioral regulation difficulties to enable study of offspring regulation in relation to multiple dimensions relevant to risk. This was done by over-selecting families where one or more of the biological parents had a past/current diagnosis or current elevated symptoms of ADHD, defined as >80^th^ percentile on the Barkley Adult ADHD Rating scale (BAARS-IV) Quick Screen (Barkley, 2011) (79% of families met these criteria). ADHD symptoms were chosen as a psychopathology proxy for dysregulation liability because ADHD has very high heritability (Larsson et al., 2014) and in addition to evidence of offspring experiencing symptoms such as impulsivity and hyperactivity, they are also likely to have difficulties in emotion regulation (Sullivan et al., 2015). We enrolled 62 women who were followed through 68 pregnancies. After enrolling in the study with the first child, six women conceived a second child and completed the same assessments for their second pregnancy, resulting in complete data on six sibling pairs. The nesting of children within families was handled in our statistical analyses (described in *Analytic Strategy*).

#### Exclusion Criteria

Exclusion criteria included high-risk or medically complicated pregnancy, extreme life circumstances (specifically, homelessness), being <18 years old, and active substance use (including alcohol, tobacco, marijuana, opioids, cocaine). Six women enrolled in the study were taking psychotropic medication during pregnancy and were excluded from the current analyses because of *a priori* concern that this medication use might confound results. In *post hoc* sensitivity analyses, we re-ran our models with these women included; the same pattern of findings emerged.

### Measures

#### Maternal Pre-pregnancy BMI

Participants’ medical records were reviewed for maternal pre-pregnancy BMI (kg/m^2^).

#### Maternal Psychological Distress

A potential alternative explanation for any inflammation related findings would be elevated maternal stress or her own NA, which could be an alternative source of inflammation or indicate potential genetic transmission of NA proneness from mother to child. At the third trimester visit, mothers completed the Center for Epidemiologic Studies Depression Scale (CES-D) (Radloff, 1977) (α = 0.92) and the Perceived Stress Scale (PSS) (Ezzati et al., 2014) (α = 0.91). Maternal scores on the CES-D and the PSS were highly correlated (*r* = 0.84), so they were converted to z scores and then averaged to create a composite variable. This variable was used as a covariate in our multivariate models. Mothers also completed the CES-D at 6 months (α = 0.94); these scores were included in sensitivity analyses.

#### Maternal Circulating FAs

Maternal circulating FAs were assessed using blood samples collected in the third trimester. Blood was drawn by venipuncture, centrifuged, and plasma was separated, aliquoted, and frozen at −80°C until assay. Plasma FAs were analyzed by direct transesterification using a Trace GC coupled to a DSQ mass spectrometer (ThermoElectron) as described previously (Lagerstedt et al., 2001). In addition to examining the total n-3 score [the sum of eicosapentaenoic acid (EPA), docosahexaenoic acid (DHA), and alpha-linolenic acid concentrations], we also separately examined the two most bioactive FAs: DHA and EPA. These two FAs are particularly important for fetal brain development (Innis, 2008; Coletta et al., 2010).

#### Maternal Ratings of Infant NA

When children were 6 months old, mothers completed the Revised Infant Behavior Questionnaire (IBQ-R) (Gartstein and Rothbart, 2003). Mothers were asked to rate how often their child completed various behaviors over the previous week on a seven-point scale (1 = *never* to 7 = *always*). In our models, we examined the Sadness (α = 0.86) and Fear (α = 0.84) subscales.

#### Observational Measures of Infant NA

The well-established still face paradigm (Tronick et al., 1979; Moore et al., 2009) was used to measure the infant’s reaction to the mother’s lack of emotional response. The mother placed the infant in a high chair and sat in a chair an arm’s length away. The mother was instructed to play with the infant for 2 min as she normally would. The mother was then instructed to turn her face away from the infant for 15 s and then turn back to face the infant for the still face period (2 min), during which the mother maintained a neutral and expressionless face and did not interact with the child. The mother was then instructed to look away again for 15 s and then return to interacting and playing with the infant as she normally would for an additional 2 min. If the infant became persistently upset, the still face period was stopped early, and the pair moved into the reunion period. Pacifiers and other toys were not allowed during this task. Five infants’ data were coded as missing because the mother interacted with the infant during the still face period; individuals who interacted with their infant during this task did not differ from the rest of the sample on any of the variables included in the current study.

Two observers, blind to the study hypotheses, coded each behavioral assessment video using a modified version of a published coding scheme (Moore et al., 2009). Infant behavior was coded in increments of 5 s for four mutually exclusive behavioral categories: affective expression, gaze, vocalization, and reactive/regulatory behavior. Infant behaviors were coded through four separate viewings of the video: one for each of the behavioral categories. In each 5 s task epoch, affective expression was coded as Positive, Negative, Neutral, or Obscure. The number of epochs rated as Negative was divided by the total number of task epochs to derive a score which captured the percentage of time the child expressed NA. Vocalization was coded as None/Not Negative or Negative. Again, the number of task epochs rated as containing negative vocalizations was divided by the total number of task epochs to derive a percentage of negative vocalizations score. Table 1 provides operational definitions for each of these behavioral categories. Infant behaviors were coded through four separate viewings of the video: one for each of the behavioral categories.

**TABLE 1:** Operational definitions for behavioral coding.

**Measure**	**Behavior**	**Description**
Affective expression	Positive	Infant’s corners of mouth raised and/or cheeks raised.
	Negative	Infant’s brows may be sharply lowered and eyes may be tightly closed. This code includes anger, sadness, and frustration.
	Obscure	Infant’s mouth or face hidden from view for the entire interval.
	Neutral	Infant displaying a relaxed face with no obvious muscle tension.
Vocalization	None/not negative	Infant is not vocalizing or vocalizations are not indicative of being fussy or upset. This code includes silence, cooing, laughing, babbling, coughing, and sneezing.
	Negative	Infant is displaying negative communication such as fussing, crying, screaming (i.e., if upset or angry), and other expressions of mild fussiness.

Inter-observer reliability was determined by analyzing percentage agreement. All of the videos were assessed for agreement between the two independent coders. The mean percentage agreement between coders was greater than 85% for all behaviors.

#### Maternal Gestational Inflammation

Maternal inflammation was assessed using blood samples collected during the third trimester of pregnancy. Plasma samples were assayed for concentrations of interleukin-6 (IL-6), tumor necrosis factor-alpha (TNF-α) and monocyte chemoattractant protein-1 (MCP-1). These cytokines were selected because they index the nuclear factor-κappa B signaling pathway (Liu et al., 2017) which has been associated with obesity (Baker et al., 2011). Sample collection and assay followed published procedures (Gustafsson et al., 2018), as follows.

Blood was drawn by one-time venipuncture into K_2_ EDTA tubes (BD Vacutainer Systems, Franklin Lakes, NJ, United States). The blood was then centrifuged at 2800 rpm for 15 min at 4°C. Plasma was separated, collected, aliquoted, and frozen at −80°C until assayed.

Plasma concentrations of IL-6 were measured by enzyme-linked immunosorbent assays (Human IL-6 quantikine HS ELISA kits (HS600B; assay range: 0.2–10 pg/ml, sensitivity: 0.11 pg/ml), R&D Systems, Inc., Minneapolis, MN, United States) according to the manufacturer’s instructions. All standards and samples were run in duplicate. Plasma samples were initially diluted 1:2 using Assay Diluent RD1-75 and incubated for 2 h at room temperature on a horizontal orbital microplate shaker. Following standard wash procedures, human IL-6 HS Conjugate was added to each well, and plates were incubated as described above. Plates were then washed and incubated with the Substrate Solution (60 min), Amplifier Solution (30 min), and Stop Solution. Plates were read within 30 min of adding the Stop Solution using a microplate spectrophotometer (Benchmark Plus microplate, Bio-Rad Laboratories, Inc., Hercules, CA, United States). Plasma concentrations of TNF-α (assay range: 5.3–3,900 pg/ml, sensitivity: 1.5 pg/ml) and MCP-1 (assay range: 32.0–23,500 pg/ml, sensitivity: 0.47 pg/ml) were assayed using Luminex polystyrene bead-based multiplex immunoassays (customized Luminex Performance Human Obesity Panel, FCST08-05; R&D Systems) according to the manufacturer’s instructions. All standards and samples were run in duplicate. Plasma samples were initially diluted 1:2 using the matrix solution provided, and samples were incubated overnight at 4°C with color-coded beads that were pre-coated with cytokine-specific capture antibodies. The plates were then washed by vacuum filtration, incubated with biotinylated detection antibodies (1 h, room temperature), washed, and incubated with phycoerythrin-conjugated streptavidin (30 min, room temperature). Plates were read on the dual-laser, flow-based Luminex 100 Analyzer (Luminex, Austin, TX, United States). For both the ELISAs and multiplex assays, sample values were determined based on standard curves calculated using computer software to generate four- and five-parameter curve-fits, respectively (Prism 7 for Windows, GraphPad Software, Inc., La Jolla, CA, United States). Cytokine values were log transformed to correct for non-normality prior to analysis, although raw cytokine values (in pg/ml) are presented in Table 2 for ease of interpretation.

**TABLE 2:** Sample demographics and descriptive statistics (*N* = 62).

	**Mean (SD) or%**	**Median (IQR)**	**Range**
Maternal age, years	30.4 (5.0)	30.0(27.1–34.0)	18–41.3
Paternal age, years	33.3 (6.3)	32.0(28.8–37.0)	23–50
Maternal education^a^	6.8 (1.2)	7.0(5.0–8.0)	5–9
Paternal education^a^	6.6 (1.6)	7.0(5.0–8.0)	3–9
Child sex,% female	40%		
% Taking omega-3 fatty acid supplement	40%		
% Breastfeeding at 6 months	94.7%		
*Child race*			
European American	76.8%		
Asian	5.4%		
African American	5.4%		
Hispanic	5.4%		
Native American	3.6%		
Biracial	3.6%		
Maternal pre-pregnancy BMI	27.2 (7.1)	25.0(23.0–30.4)	17–49
*Maternal omega–3 fatty acids*			
Total Omega-3 fatty acids (nmol/ml)	518.6 (170.9)	524.0(385.1–632.6)	204.6–859.1
DHA (nmol/ml)	331.0 (116.5)	316.7(243.8–425.9)	131.2–559.7
EPA (nmol/ml)	50.0 (44.7)	37.7(25.9–54.1)	16.9–250
*Maternal psychological distress*			
Maternal perceived stress	15.6 (7.5)	15.5(8.0–22.0)	3–31
Maternal depressive symptoms	15.7 (11.2)	13.0(7.4–21.0)	0–53
*Infant negative affect*			
Still face paradigm negative behavior	21.2 (26.2)	10.0(0–44.5)	0–88
Still face paradigm negative vocalizations	22.8 (32.8)	8.0(0–39.8)	0–100
IBQ–R sadness	3.7 (0.9)	3.6(3.0–4.5)	2.1–5.2
IBQ-R fear	2.4 (0.7)	2.4(1.8–2.9)	1.3–3.8
*Maternal inflammation*			
IL-6 (pg/ml)	1.6 (0.8)	1.5(1.1–2.0)	0.4–3.6
TNF-α (pg/ml)	11.3 (3.2)	10.8(8.9–13.5)	5.7–17.9
MCP-1 (pg/ml)	93.0 (26.5)	88.4(78.0–113.0)	26.4–171.5

*^*a*^1 = Grade School, 2 = Some High School, 3 = High School Equivalent, 4 = High School degree, 5 = Some College but no degree, 6 = Associates degree, 7 = Bachelor’s degree, 8 = Masters, Law, 2–3 years degree, 9 = Doctorate, Ph.D., Medical degree. BMI, body mass index; DHA, docosahexaenoic acid; EPA, eicosapentaenoic acid; IBQ-R, Revised Infant Behavior Questionnaire. IL-6, interleukin-6; TNF-α, tumor necrosis factor-alpha; MCP-1, monocyte chemoattractant protein-1. At the 3rd trimester visit, women self-reported all supplements that they were consuming, including supplements that contain omega-3 fatty acids. At 6 months postpartum, women reported whether they were currently breastfeeding their child.*

Prior to conducting our sensitivity analyses, we conducted a confirmatory factor analysis (CFA) to examine whether it was appropriate to consider IL-6, TNF-α, and MCP-1 values together as a latent variable (simultaneously considering multiple indices of maternal inflammation—and in this case multiple indices of the NF-κB signaling pathway–in this manner is intended to yield a more reliable and comprehensive assessment of maternal systemic inflammation than any single cytokine value). Results from this CFA (results available by request from the authors) supported the construction of a latent variable indicated by the three pro-inflammatory cytokines, IL-6, TNF-α, and MCP-1; this latent variable was used in all supplemental inflammation models.

### Analytic Strategy

Our research questions were tested using M*plus* 7.4 (Muthén and Muthén, 1998–2017) and the robust maximum likelihood estimator which can accommodate non-normal data. Missing data were handled using full information maximum likelihood, a missing data technique that uses all available information to produce estimates that are less biased and more efficient than those produced via other methods of handling data that are missing at random (Collins et al., 2001). Auxiliary variables were included to maximize precision of the missing data matrix (Graham, 2003). These auxiliary variables were maternal and paternal ADHD symptoms (assessed using the BAARS-IV Quick Screen), (Barkley, 2011) age (years), and education (highest completed; 1 = *Grade School*, 9 = *Doctorate*). Non-independence of observations (i.e., the nesting of children within families) was handled using the M*plus Cluster* command.

We first estimated a series of univariate models wherein each infant behavioral measure was regressed on (a) maternal pre-pregnancy BMI, (b) total n-3s, (c) DHA, and (d) EPA. Each infant behavior measure was considered in its own model. Uncorrected *p*-values are reported for ease of comparison with other studies and because corrected *p*-values elevate Type II error in this small study; however, family-wise error was controlled and adjusted *p-*values reported also. A Benjamini-Hochberg correction (Benjamini and Hochberg, 1995) was applied to account for multiple comparisons due to moderate correlation among outcomes tested.

Next, we estimated a series of regression analyses where maternal pre-pregnancy BMI and each measure of circulating FAs were considered in the same model, while simultaneously controlling for maternal third trimester psychological distress.

To test whether maternal n-3 FAs moderated the effect of maternal pre-pregnancy BMI on infant NA, we added an interaction term which captured the interaction between maternal BMI and the particular measure of FAs to the previous model. Based on our theoretical interest in factors that might ameliorate the effect of BMI on infant outcomes we *a priori* decided to examine moderation only for infant behavioral measures that were associated with BMI in the univariate models. In these models, each infant behavior measure was regressed on maternal pre-pregnancy BMI, the particular measure of maternal FAs, maternal psychological distress, and the interaction between maternal BMI and circulating FAs. All continuous variables were mean-centered prior to model estimation.

Omega-3 fatty acid levels were treated as a continuous variable in all analyses. However, to visualize significant interaction effects, we employed the pick-a-point method and probed this interaction using methods described by Preacher et al. (2006). The pick-a-point approach involves selecting three values of your moderator and plotting the model-implied regression lines for these three values, as a way to visually illustrate the nature of the interaction.

### Analyses Examining Inflammation

As described in the “Introduction,” section an explicit test of inflammation as a mechanism through which BMI and FA interactively influence infant NA was outside of the scope of this study. However, we did conduct the following analyses, to provide additional context for our findings and preliminary proof of concept.

First, we examined whether, in this sample, maternal BMI and FA levels were associated with maternal inflammation during pregnancy. In separate models, we regressed the maternal third trimester inflammation latent variable on (a) maternal pre-pregnancy BMI, (b) total n-3s, (c) DHA, and (d) EPA. Next, we examined whether maternal BMI and fatty acid levels interacted to predict maternal inflammation. Specifically, we regressed maternal inflammation on maternal BMI, fatty acid levels, and an interaction term that captured the interaction between the particular measure of FAs (again, total n-3s, DHA, and EPA were examined in separate models). All continuous variables were mean-centered prior to model estimation. Significant interactions were probed as above.

In a separate set of univariate models, aimed at examining whether maternal inflammation was related to infant NA, we regressed each infant behavioral measure on maternal inflammation.

## Results

### Descriptive Statistics

Demographic data about this sample as well as means (SDs) and medians (interquartile range) for the study variables appear in Table 2. None of the demographic variables presented in this table were significantly correlated with infant NA (*p*s > 0.06) and thus were not included as covariates in analyses.

### Relating BMI, Fatty Acids, and Infant NA

Table 3 presents the results from the univariate models in which each measure of infant NA was successively regressed on maternal pre-pregnancy BMI and each measure of circulating FAs. Increased maternal pre-pregnancy BMI was associated with greater infant negative behavior during the still face paradigm (β = 0.45, corrected *p* = 0.016). Additionally, EPA was associated with less mother-reported sadness (β = −0.56, corrected *p* < 0.001) and fear (β = −0.47, corrected *p* < 0.001), as well as less negative behavior (β = −0.38, corrected *p* = 0.004) and negative vocalizations (β = −0.36, corrected *p* = 0.004) during the still face paradigm. DHA and the total n-3 FA score were not significantly associated with infant NA.

**TABLE 3:** Results of univariate models where each infant negative affect measure was regressed on maternal pre-pregnancy BMI and omega-3 fatty acid concentrations.

	**IBQ-R sadness**			**IBQ-R fear**			**Still face paradigm negative behavior**			**Still face paradigm negative vocalizations**		
	**β(SE)**	***p*-value**	**B-H**	**β(SE)**	***p-*value**	**B-H**	**β(SE)**	***p-*value**	**B-H**	**β(SE)**	***p*-value**	**B-H**
			**corrected**			**corrected**			**corrected**			**corrected**
			***p-*value**			***p-*value**			***p* value**			***p-*value**
Pre-pregnancy BMI	0.06 (0.15)	0.682	0.712	−0.10 (0.17)	0.566	0.669	0.45 (0.16)	0.004	0.016^∗^	0.34 (0.20)	0.095	0.222
Omega-3 fatty acids	−0.36 (0.19)	0.064	0.170	−0.28 (0.18)	0.111	0.222	−0.17 (0.16)	0.282	0.404	−0.18 (0.17)	0.286	0.404
DHA	−0.32 (0.17)	0.052	0.156	−0.21 (0.17)	0.216	0.370	−0.09 (0.17)	0.585	0.669	−0.08 (0.19)	0.670	0.712
EPA	−0.56 (0.12)	0.000	< 0.001^∗∗^	−0.47 (0.12)	0.000	< 0.001^∗∗^	−0.38 (0.12)	0.001	0.004^∗∗^	−0.36 (0.11)	0.001	0.004^∗∗^

*^∗^*p* < 0.05, ^∗∗^*p* < 0.01. B-H corrected *p*-value = Benjamini-Hochberg corrected *p*-value. BMI, body mass index; DHA, docosahexaenoic acid; EPA, eicosapentaenoic acid; IBQ-R, Revised Infant Behavior Questionnaire.*

### Independent Effects of BMI and FAs, Covarying for Maternal Psychological Distress

These associations remained similar when maternal pre-pregnancy BMI was considered in a model with each measure of circulating FAs and with maternal psychological distress during the third trimester (Table 4). Maternal pre-pregnancy BMI remained significantly associated with infant negative behavior during the still face after covarying for maternal psychological distress and total n-3 FAs (β = 0.39, *p* = 0.027) as well as psychological distress and DHA (β = 0.36, *p* = 0.036) but was no longer significant when considered in a model with EPA and psychological distress (β = 0.31, *p* = 0.140), though the effect size was similar in magnitude. Maternal EPA was associated with maternal rated infant sadness (β = −0.61, *p* < 0.001) and fear (β = −0.53, *p* < 0.001) as well as infant negative vocalizations during the still face paradigm (β = −0.30, *p* = 0.037).

**TABLE 4:** Results from multivariate models that consider both pre-pregnancy BMI and Omega-3 fatty acid concentrations, and covary for maternal psychological distress.

	**IBQ-R sadness**		**IBQ-R fear**		**Still face paradigm negative behavior**		**Still face paradigm negative vocalizations**	
	**β(SE)**	***p*-value**	**β(SE)**	***p*-value**	**β(SE)**	***p*-value**	**β(SE)**	***p*-value**
Pre-pregnancy BMI	−0.13 (0.15)	0.401	−0.28 (0.19)	0.130	0.39 (0.14)	0.027^∗^	0.40 (0.22)	0.065
Omega-3 fatty acids	−0.32 (0.20)	0.107	−0.30 (0.17)	0.081	0.02 (0.16)	0.883	−0.09 (0.18)	0.599
Maternal psychological distress	0.27 (0.13)	0.041	0.29 (0.15)	0.048	0.24 (0.14)	0.084	−0.22 (0.15)	0.123
Pre-pregnancy BMI	−0.05 (0.14)	0.722	−0.22 (0.17)	0.187	0.36 (0.17)	0.036^∗^	0.39 (0.21)	0.068
DHA	−0.29 (0.17)	0.091	−0.19 (0.16)	0.225	0.04 (0.18)	0.815	−0.04 (0.20)	0.831
Maternal psychological distress	0.25 (0.13)	0.056	0.30 (0.15)	0.041	0.26 (0.14)	0.066	−0.20 (0.15)	0.167
Pre-pregnancy BMI	−0.20 (0.13)	0.114	−0.33 (0.15)	0.026	0.31 (0.19)	0.140	0.33 (0.22)	0.142
EPA	−0.61 (0.13)	< 0.001^∗∗^	−0.53 (0.12)	< 0.001^∗∗^	−0.21 (0.14)	0.093	−0.30 (0.14)	0.037^∗^
Maternal psychological distress	0.27 (0.12)	0.025	0.28 (0.13)	0.031	0.21 (0.13)	0.110	−0.22 (0.14)	0.118

*^∗^*p* < 0.05, ^∗∗^*p* < 0.01. BMI, body mass index; DHA, docosahexaenoic acid; EPA, eicosapentaenoic acid; IBQ-R, Revised Infant Behavior Questionnaire.*

In sensitivity analyses, we examined whether these effects survived controlling for maternal depressive symptoms at 6 months (included in the model in place of maternal third trimester psychological distress). Each of these effects survived this control.

### Moderation Analyses

The results from the moderation analyses appear in Table 5. Based on our *a priori* decision to only investigate moderation for infant outcomes that were associated with maternal BMI in bivariate analyses, these models were focused on infant negative behavior during the still face paradigm.

**TABLE 5:** Results of moderation analyses investigating the interaction between maternal pre-pregnancy BMI and omega-3 fatty acids in the prediction of infant negative affect.

	**Still face paradigm**	
	**negative behavior**	
	**β(SE)**	***p*-value**
Pre-pregnancy BMI	0.33 (0.14)	0.018^∗^
Omega-3 fatty acids	−0.13(0.14)	0.380
Maternal psychological distress	0.07 (0.17)	0.663
BMI × omega-3 fatty acids	−0.41(0.15)	0.007^∗∗^
Pre-pregnancy BMI	0.29 (0.11)	0.009^∗∗^
Third trimester DHA	−0.09(0.15)	0.557
Maternal psychological distress	0.16 (0.14)	0.259
BMI × DHA	−0.42(0.14)	0.002^∗∗^
Pre-pregnancy BMI	−0.02(0.23)	0.016^∗^
Third trimester EPA	−0.80(0.33)	0.922
Maternal psychological distress	0.17 (0.13)	0.183
BMI × EPA	−0.56(0.22)	0.012^∗^

*^∗^*p* < 0.05, ^∗∗^*p* < 0.01. BMI, body mass index; DHA, docosahexaenoic acid; EPA, eicosapentaenoic acid.*

**FIGURE 1:**
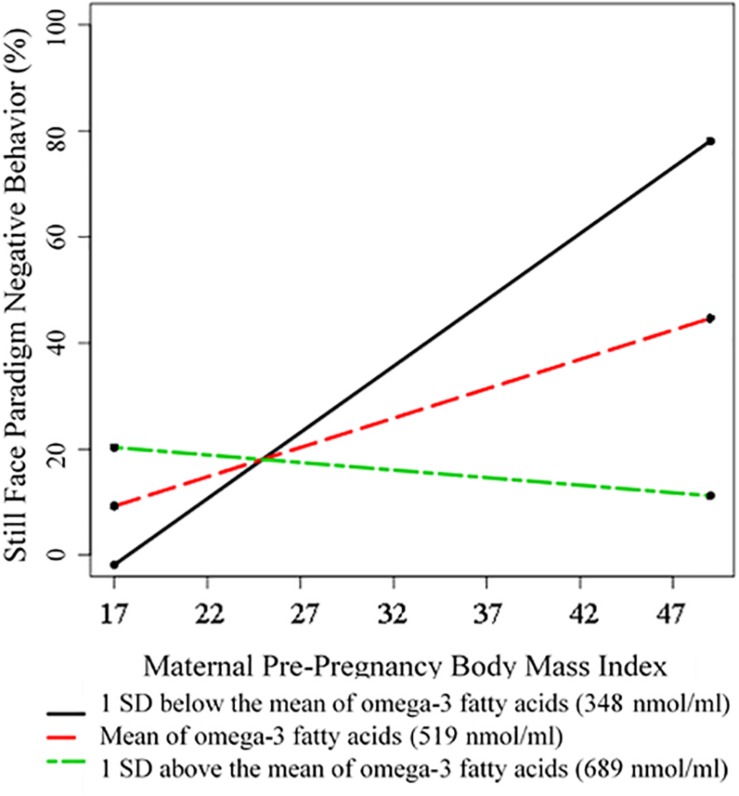
Visual Depiction of the Interaction between Maternal Pre-Pregnancy BMI and Omega-3 Fatty Acids in Predicting Infant Negative Behavior during the Still Face Paradigm. Omega-3 fatty acid levels were treated as a continuous variable in all analyses. However, for the purposes of visualization, we employed the pick-a-point method and probed this interaction following the direction of Preacher et al. (2006). The pick-a-point approach involves selecting three values of your moderator and plotting the model-implied regression lines for these three values, as a way to visually illustrate the nature of the interaction (it does not affect the moderation analyses, where levels of omega-3 fatty acid concentrations were treated as a continuous variable). Following convention, in this figure we have plotted the model-implied slopes that correspond to our sample mean, one standard deviation above our sample mean, and one standard deviation below our sample mean of circulating omega-3 fatty acid concentrations.

Results suggest that n-3 FAs moderate the effect of maternal pre-pregnancy BMI on infant negative behavior during the still face paradigm. As is visually depicted in Figure 1, for individuals who had average levels of circulating n-3 FAs (defined as our sample mean), there was a positive association between increased maternal pre-pregnancy BMI and greater infant NA (β_Maternal Pre–pregnancy BMI_ = 0.33, *p* = 0.02). However, the BMI-infant NA association was weaker for individuals who had higher circulating levels of n-3 FAs (β_interaction_ = −0.41, *p* = 0.007). This is supportive of our hypothesis that n-3 FAs may buffer children against the negative impact of increased maternal pre-pregnancy BMI. See Figure 1 for a visual depiction of this finding. The models that considered EPA and DHA separately yielded the same pattern of results, suggesting that this moderation effect was not specific to only one of these n-3 FAs.

### Inflammation Analyses: Preliminary Proof of Concept

As expected, increased maternal pre-pregnancy BMI was associated with greater maternal inflammation during the third trimester of pregnancy (β = 0.88, *p* < 0.001), while total n-3 FAs (β = −0.48, *p* = 0.021), EPA (β = −0.32, *p* = 0.011), and DHA (β = −0.47, *p* = 0.008) were associated with less maternal inflammation. Further, maternal pre-pregnancy BMI and total n-3 FA levels interacted to predict maternal inflammation (β = −0.39, *p* = 0.026); the positive association between increased maternal pre-pregnancy BMI and maternal inflammation is strongest for women who have low n-3 FA levels, and is weaker for those who have higher levels. The maternal BMI by DHA interaction term (*p* = 0.529) and the BMI by EPA interaction term (*p* = 0.270) were not statistically significant (these were considered in separate models), suggesting that total n-3 FAs are more influential in this context.

Maternal inflammation was significantly associated with maternal report of infant sadness (β = 0.88, *p* < 0.001), but was not significantly associated with the other measures of infant NA (*p*s > 0.411).

## Discussion

The goal of the current study was to examine whether maternal pre-pregnancy BMI and circulating n-3 FAs during the third trimester of pregnancy independently and/or interactively predicted infant NA, an early risk factor for psychopathology. Though increased maternal prenatal adiposity and n-3 FA intake have each been linked with psychopathology in older children, this study’s focus on an infant marker of such risk is novel and has potential to inform our understanding of the developmental pathways through which gestational factors influence long-term risk. Study results suggest that there are both independent and interactive associations. To our knowledge, this is the first human study to report evidence that n-3 FAs may modify the effect of increased adiposity on infant outcomes.

Results from our univariate models suggest that greater maternal pre-pregnancy BMI is associated with greater observer ratings of infant NA, a finding that is consistent with our research in non-human primates (Thompson et al., 2017, 2018). Additionally, we found that greater EPA concentrations are associated with less NA, as rated by both mothers and independent observers. This same general pattern of findings emerged when pre-pregnancy BMI and n-3 FAs were included in the same model along with maternal third trimester psychological distress, a potential confounder of this association. However, several of these effects did not survive correction for multiple comparisons. The protective effect of EPA on maternal report of infant NA (both fear and sadness) appears to be the most robust of these associations. Omega-3 FAs are important during pregnancy, as they support optimal placental functioning and are required for healthy fetal growth and brain development, particularly during the third trimester (Greenberg et al., 2008; Chavan-Gautam et al., 2018). Their protective effects on fetal growth and child cognitive development have been well-documented (Innis, 2007; Greenberg et al., 2008), though this is the first study to link maternal n-3 FA concentrations during pregnancy with measures of infant emotional development.

Interestingly, EPA emerged as a particularly consistent predictor of infant NA, as compared to either DHA or total n-3 FAs. Specifically, mothers who had high levels of circulating EPA tended to have children who cried less and were less fearful and sad. Much of the existing literature examining the effects of n-3 FAs on offspring development has focused on the effects of DHA; though, as mentioned above, they have largely investigated cognitive, rather than emotional, correlates of maternal nutrition. Previous research examining the efficacy of n-3 supplementation for the treatment of adult depression suggests that EPA may be more efficacious for improving depressive symptoms than DHA (Parker et al., 2006; Martins, 2009). Infant NA is an early life risk factor for depression; thus maternal EPA consumption may be an important protective factor for offspring emotional health.

The current study also found evidence of interactive effects, such that the effect of maternal pre-pregnancy BMI on observer ratings of infant NA was moderated by maternal n-3 FA concentrations during pregnancy. Specifically, we found that the association between increased maternal pre-pregnancy BMI and increased observer ratings of infant NA was weaker for women who had higher circulating n-3 FA concentrations. That is, higher n-3 FAs appear to buffer children against the negative consequences of increased maternal pre-pregnancy BMI. These effects do not appear to be specific to a particular type of n-3 FA as we observed the same pattern of findings when examining DHA and EPA. In our preliminary proof of concept analyses, we found evidence that this reduction in risk may be due to the interactive effect of maternal BMI and n-3 FAs on maternal inflammation during the third trimester. Specifically, we found that the association between increased maternal BMI and increased infant NA was weaker when women had higher n-3 FA levels; greater maternal prenatal inflammation, in turn, was association with greater infant NA. These findings, if replicated, suggest that n-3 supplementation during pregnancy may help reduce children’s NA early in life (and by extension, reduce their risk for later psychopathology), particularly among children whose mothers are overweight or obese. Omega-3 supplementation is a promising intervention target as it is safe and easier to achieve adherence with than weight loss or dietary modifications, which are often ineffective (Jeffery et al., 2000; Mann et al., 2007). Moreover, weight loss is not typically recommended during pregnancy even among obese women (Kapadia et al., 2015), making this a less promising avenue for intervention.

This study had several strengths. First, utilizing prospective longitudinal data, this is one of the first human studies to examine the effects of increased maternal adiposity on infant emotional development. Though previous research has linked increased maternal prenatal adiposity with risk for psychopathology in older children, few studies have considered these factors in relation to infant markers of risk. Additionally, as described above, the existing literature examining the effects of maternal n-3 FAs on child development has focused on child cognition or motor development, making our assessment of infant emotional development unique. Methodological strengths included consideration of multiple assessment methods for infant NA as well as our inclusion of a measure of circulating n-3 FAs obtained from plasma samples (which yields a more objective measure of maternal dietary intake than self-report measures, and more accurately reflects fetal exposure to n-3 FAs).

This study also had limitations. The present findings are preliminary due to the small sample size. Though our use of longitudinal data was a strength of this study, we only examined three time points of data. Future research should incorporate additional prenatal and postnatal assessments. Additionally, we utilized data from a unique sample of women that was over-represented for women prone to emotional dysregulation (i.e., those with a diagnosis of ADHD or endorsing high levels of ADHD symptoms were over-sampled), and although this increased our power to detect effects, it also leaves us unable to generalize these findings to a general population sample. However, this study does justify larger studies. Although we have strong reason to believe that increased maternal adiposity and n-3 FAs may influence the developing child via their effect on the *in utero* inflammatory profile, the current study did not explicitly test for mediation or moderated-mediation (as would be necessary to test this hypothesis), nor did it assess inflammation in amniotic fluid, which might yield a more direct measure of child cytokine exposure. This study’s relatively small sample size limited our ability to test such a complicated model, though our supplemental inflammation analyses provide preliminary proof of concept and support that this may be the mechanism of influence. Specifically, we found that the effect of maternal pre-pregnancy BMI on maternal inflammation was strongest for women who had lower levels of n-3 FAs and was weaker for women who had higher levels of n-3 FA levels. Additionally, we found that maternal inflammation was associated with maternal report of infant sadness, though it was not related to the other measures of infant NA. These null findings may reflect a lack of power to detect such an effect, differences in the context and the types of NA-related behaviors that maternal and laboratory observations capture, or the numerous potential contributors by which maternal obesity influences infant neurobehavioral development including alterations in cytokines not examined in the current study, exposure to increased nutrients such as glucose and saturated fatty acids, and alterations in metabolic hormones (Rivera et al., 2015). Though preliminary, we believe that these results complement the results of previous research that has shown that fatty acids can, directly and indirectly, influence the expression of adipokines (cytokines secreted by adipose tissue), as well as the production of cytokines from other cell types (Drevon, 2005). Further, these results are in alignment with *in vitro* experiments with human macrophages and hepatocytes which indicate that EPA can reduce cytokine expression in the context of induced inflammatory response (Hao et al., 2010). Future research will need to investigate and clarify such biological mechanisms as they relate to the current findings.

In summary, the current study is the first human study to investigate the independent and interactive effects of maternal pre-pregnancy BMI and circulating n-3 FAs during pregnancy on infant NA, an early life risk factor for psychopathology. Results indicate that EPA has a particularly robust association with decreased infant fear and sadness according to maternal report. Further, we provide novel evidence that n-3 FAs during pregnancy may modify the effects of increased maternal pre-pregnancy BMI on observer ratings of infant NA. Specifically, we found that n-3 FAs buffer infants against the negative consequences of increased maternal adiposity.

## Data Availability Statement

The datasets generated for this study are available on request to the corresponding author.

## Ethics Statement

The studies involving human participants were reviewed and approved by the Oregon Health & Science University Institutional Review Board. Written informed consent to participate in this study was provided by the participants and/or the participants’ legal guardian/next of kin.

## Author Contributions

ES and JN conceived the project. HG, ES, JN, KH, and JL designed the research. AA, EN, and CS performed the experiments. HG, JN, and ES analyzed the data. All authors discussed the data. HG and ES wrote the manuscript, with contributions from all other authors.

## Conflict of Interest

The authors declare that the research was conducted in the absence of any commercial or financial relationships that could be construed as a potential conflict of interest.
